# Regulation of proteinaceous effector expression in phytopathogenic fungi

**DOI:** 10.1371/journal.ppat.1006241

**Published:** 2017-04-20

**Authors:** Kar-Chun Tan, Richard P. Oliver

**Affiliations:** Centre for Crop and Disease Management, Department of Environment and Agriculture, Curtin University, Bentley, Western Australia, Australia; Scottish Crop Research Institute, UNITED KINGDOM

## Abstract

Effectors are molecules used by microbial pathogens to facilitate infection via effector-triggered susceptibility or tissue necrosis in their host. Much research has been focussed on the identification and elucidating the function of fungal effectors during plant pathogenesis. By comparison, knowledge of how phytopathogenic fungi regulate the expression of effector genes has been lagging. Several recent studies have illustrated the role of various transcription factors, chromosome-based control, effector epistasis, and mobilisation of endosomes within the fungal hyphae in regulating effector expression and virulence on the host plant. Improved knowledge of effector regulation is likely to assist in improving novel crop protection strategies.

## Introduction

The host—fungus interface can be considered a biological battlefield as both organisms attempt to outwit each other [1]. The host must recognise the pathogen so as to initiate an effective defence response, whereas the pathogen must coordinate its pathogenicity arsenal so as to enable the colonisation of the host. For instance, some genes within the fungal pathogen are up-regulated to subvert host metabolism by detoxifying defence compounds [2], to subdue the host immune system [3], and to assimilate nutrients during colonisation [4]. In addition, some genes must be down-regulated to avoid host recognition. For example, the anthracnose pathogen *Colletotrichum graminicola* down-regulates the expression of β-1,6-glucan biosynthesis genes to attenuate pathogen-associated molecular pattern-triggered immunity during biotrophic infection of maize [5].

Effectors can be defined as secreted molecules from a microbe that modulate an interaction with a host [6]. Avirulence (Avr) and necrotrophic effectors ([NEs], also known as host-selective toxins) are the major classes of host-specific effectors. Avr effectors are typically (but not exclusively) associated with biotrophic pathogens where recognition is conducted by host resistance (R) proteins. Recognition results in an immune response from the host leading to resistance. In the absence of the R protein recognition, Avr effectors may assist in virulence by manipulating the host immune system to the pathogen’s favour in a process known as effector-triggered susceptibility [7]. NEs function as pathogenicity factors that induce tissue death on host plants that possess a dominant sensitivity/susceptibility genotype [1]. NEs are exclusively associated with fungal pathogens that exhibit a necrotrophic lifestyle. The role of fungal effectors in host virulence has been comprehensively reviewed elsewhere [6,8]. This review highlights studies on transcription factors (TFs) and processes affecting expression of genes that encode proteinaceous effectors in phytopathogenic fungi (Table 1).

**Table 1:** A summary of characterised fungal effector genes and their regulatory information.

Organism	Effector gene or necrosis/chlorosis-inducing activities	Mode of regulation*	Reference
*Cladosporium fulvum*	*Avr2*	*CfWor1* (+)	[57]
	*Avr4E*	*CfWor1* (+)	[57]
	*Avr4*	*CfWor1* (+)	[57]
	*Avr9*	Nitrogen (-)	[19]
		*Nrf1* (+)	[22]
		*CfWor1* (+)	[57]
	*Ecp6*	*CfWor1* (+)	[57]
*Fusarium oxysporum f*. *sp lycopercisi*	*SIX1*	*SGE1* (+)	[54]
	*SIX3*	*SGE1* (+)	[54]
	*SIX5*	*SGE1* (+)	[54]
*Leptosphaeria maculans*	*AvrLm1*	*LmStuA* (+)	[51]
		*LmHP1* (-)	[82]
		*LmDIM5* (-)	[82]
	*AvrLm3* (*-Rlm3*)^	*AvrLm4-7* (-)	[44]
	*AvrLm4-7*	*LmStuA* (+)	[51]
		*LmHP1* (-)	[82]
		*LmDIM5* (-)	[82]
	*AvrLm6*	*LmStuA* (+)	[51]
*Parastagonospora nodorum*	*SnToxA*	*PnPf2* (+)	[32]
	*SnTox3*	*PnPf2* (+)	[32]
		*SnStuA* (+)	[43]
		*SnTox1* (-)	[88]
*Pyrenophora tritici-repentis*	Chlorosis-inducing factor/disease	*ToxA* (-)	[89]
	*ToxA*	*PtrPf2* (+)	[32]
*Ustilago maydis*	*Cmu1*	Endosome motility (+) and *Crk1* (-)	[91]
		*Ros1* (-)	
	*Pep1*	Endosome motility (+)	[91]
	*Pit2*	Endosome motility (+)	[91]
		*Ros1* (-)	
	*See1*	*Ros1* (+)	[58]
*Verticillium dahliae*	*Ave1*	*VdSge1* (+)	[55]

*Positive (+) or negative (-) regulator of gene expression/effector activity.

**^** It is only known that AvrLm4-7 modulates the AvrLm3–*Rlm3* interaction rather than *AvrLm3* at this stage.

## Regulation by TFs

Analysis of fungal genomes has identified at least 36 fungal TF Pfam families [9]. TFs in plant pathogenic fungi have been subjected to several comprehensive functional studies to elucidate their roles in phytopathogenicity [10,11,12,13]. Thus far, 12 TFs from four families have been characterised for their roles in the regulation of effector or candidate effector gene expression.

### Zinc-finger

Zinc (Zn)- binding proteins make up the largest family of transcriptional regulators in fungi [11,13]. Proteins in the family harbour at least one “finger-like” structure that consists of Zn bound by cysteine (Cys) or histidine residues [14]. A key function of the Zn-finger is to bind to DNA, thereby controlling transcription [15].

Zn-finger TFs were implicated in effector gene regulation in studies initiated almost two decades ago on Avr effectors of the tomato leaf mould fungus *Cladosporium fulvum* [16]. *Avr9* encodes a small Cys-rich peptide that is recognised by the host *Cf9* R gene [17]. Aside from its role as an Avr determinant, the biological function of Avr9 is not known, although it structurally resembles a carboxypeptidase inhibitor [18]. *Avr9* is highly expressed during infection but not under in vitro conditions that are nitrogen replete [19]. Analysis of the *Avr9* promoter region identified several copies of (TA)GATA, which is associated with the binding site of positive acting global nitrogen (N) regulatory proteins AreA of *Aspergillus nidulans* and NIT-2 of *Neurospora crassa*. AreA and NIT-2 are GATA TFs that possess 4-Cys Zn-fingers [20]. Mutants that carry *AreA* and *NIT-2* inactivation cannot assimilate secondary N sources, such as nitrates and various amino acids [21]. The *AreA*/*NIT-2* ortholog *Nrf1* was eventually cloned from *C*. *fulvum* [22]. The introduction of *Nrf1* into the loss-of-function *areA A*. *nidulans* restored growth on secondary N sources. Furthermore, *Nrf1* was required for N-deprivation induction of *Avr9* [22].

At this stage, it is not clear whether N limitation is an accurate reflection of the nutritional status during infection [23]. Several lines of experimental evidence indicate otherwise. For instance, the expression of other *C*. *fulvum* effector genes is not subjected to regulation by N [24]. It was also observed that during a compatible interaction between *C*. *fulvum* and tomato, the concentration of most amino acids including the nonprotein amino acid γ-aminobutyric acid (GABA) increased in the leaf apoplast [25]. It was hypothesised that GABA is a key N source in planta [26,27]. Furthermore, mutations in N assimilatory genes across many fungal pathogens do not greatly affect virulence [e.g., 22,28,29].

A central role for a Zn-finger TF in effector expression was suggested in a study on *Alternaria brassicicola*, a necrotroph that causes dark leaf spot on Brassicaceae [30]. A fungal-specific Zn_2_Cys_6_ binuclear cluster domain TF called AbPf2 was characterised for its role in virulence [31]. Mutants deleted in *AbPf2* were unable to cause lesions on *Brassica napus* and *Arabidopsis thaliana*. Transcriptional analysis of the *abpf2* mutants identified 33 down-regulated genes that code for secreted proteins. Of these, eight possess effector hallmarks such as Cys-rich, small molecular weight and the presence of signal peptides for secretion [31]. Blast analysis revealed that AbPf2 orthologues are restricted to the Pleosporales. This class includes two major fungal pathogens of wheat: *Parastagonospora nodorum*—the causal agent of septoria nodorum blotch—and the tan spot fungus *Pyrenophora tritici-repentis* [32].

*P*. *nodorum* uses a series of proteinaceous NEs to cause tissue chlorosis/necrosis on wheat cultivars that possess matching dominant susceptibility genes. Three NEs have been identified to the gene level and characterised for their role in virulence—SnToxA, SnTox1, and SnTox3. These effectors cause necrosis on wheat cultivars that carry *Tsn1* [33,34], *Snn1* [35], and *Snn3* [36,37] dominant susceptibility genes, respectively. The *P*. *nodorum*-wheat pathosystem is also governed by other effector—host dominant sensitivity gene interactions [38,39,40]. Thus far, genes encoding for the effectors and their matching host genes have yet been identified. *SnToxA*, *SnTox1*, and *SnTox3* are highly expressed during early infection [32]. This is not surprising considering that the pathogen must rapidly disable the host for invasion. Expression analysis of the *AbPf2* orthologue *PnPf2* revealed a similar expression pattern to the three effector genes. Functional analysis demonstrated that PnPf2 functions as a positive regulator of *SnToxA* and *SnTox3* expression but not *SnTox1*. Consequently, *pnpf2* mutants are nonpathogenic on wheat carrying only *Tsn1* and *Snn3* but unaffected on *Snn1* wheats [32]. The role of Pf2 was similarly examined in *P*. *tritici-repentis*. Like *P*. *nodorum*, *P*. *tritici-repentis* possesses a near-identical copy of *ToxA* that was thought to have been acquired from *P*. *nodorum* via a horizontal gene transfer event [33,41]. Like *SnToxA*, the expression of *P*. *tritici-repentis ToxA* is maximal during early infection but it is also highly expressed in vitro. Deletion of *PtrPf2* resulted in down-regulation of *ToxA* and loss of virulence on *Tsn1* wheat [32]. Thus, the Pf2 TF family possesses a conserved role in the regulation of an effector that is common between two closely related fungi that possess a necrotrophic lifestyle on wheat.

### APSES

The Asm1, Phd1, Sok2, Efg1, and StuA (APSES) family consists of basic helix-loop-helix TFs that are associated with the regulation of fungal development [42]. The APSES TF family is unique to fungi. In *P*. *nodorum*, the role of SnStuA in fungal development and virulence on wheat was examined using gene deletion strains [43]. Mutants deleted in *SnStuA* showed abnormal vegetative growth, abolished sporulation, and displayed major alterations in central carbon metabolism and were nonpathogenic on wheat. When the *snstuA* mutants were grown under in vitro conditions that are conducive to effector production, the expression of *SnTox3* was significantly reduced [43].

Seven Avr effectors have been identified and characterised from the canola blackleg fungus *Leptosphaeria maculans*: AvrLm1, AvrLm2, AvrLm3, AvrLm4-7, AvrLm6, AvrLm11, and AvrLmJ1 [44,45,46,47,48,49,50]. The *L*. *maculans* StuA TF was examined for its role in effector gene regulation in addition to virulence and vegetative development [51]. *LmStuA* knockdown via RNAi caused developmental abnormalities associated with vegetative growth and sporulation. All *lmstuA* mutants failed to cause disease on a highly susceptible *B*. *napus* cultivar. Furthermore, the expression of *AvrLm4-7*, *AvrLm1*, and *AvrLm6* was abolished in the *lmstuA* mutants when sampled during the infection period where peak expression of effector genes in the wild type (WT) was observed [51]. It is possible that the reduction in effector gene expression may be attributed to indirect regulation caused by pleiotropic effects from the perturbation of StuA function in *P*. *nodorum* and *L*. *maculans*.

### WOPR

*Candida albicans* is an opportunistic fungal pathogen of humans. The pathogen possesses a dimorphic life cycle switching from opaque yeast-like growth to filamentous form during infection. White-opaque regulator 1 (*Wor1*) was identified and characterised as a master regulator of white-opaque switching in *C*. *albicans* [52]. Wor1 belongs to the Pac2 and Ryp1 (WOPR) family [53]. The WOPR family consists of two dissimilar but highly conserved globular DNA binding domains located at the protein N-terminal [53]. *Wor1* orthologues have been identified in several phytopathogenic fungi. Functional characterisation has identified unique roles in host virulence and regulation of effectors or effector-like molecules [54,55,56,57,58].

The tomato wilt disease fungus *Fusarium oxysporum* f. sp. *lycopersici* secretes a number of small proteins into the host xylem upon infection (Six) [59]. Several studies have shed some light on the role that these proteins play in the *Fusarium*—tomato pathosystem. The *Wor1* orthologue *S**IX* *G**ene* *E**xpression 1* (*SGE1*) was originally identified through an insertional mutagenesis screen for nonpathogenic mutants [60]. Functional analyses indicate that Sge1 is localised in the nucleus and plays an essential role in virulence on tomato [54]. Gene expression analysis indicates that it functions as a positive regulator of *SIX1*, *SIX2*, *SIX3*, *and SIX5* under conditions that mimic host infection [54]. *SIX1* (*AVR3*) and *SIX3* (*AVR2*) encode proteinaceous Avr effectors that are recognised by *I-3* and *I-2* host-mediated resistance, respectively [61,62]. Six5, however, functions in conjunction with Six3 to activate *I-2*-mediated resistance [63]. The function of *SIX2* remains unclear.

In the vascular wilt pathogen of dicots *Verticillium dahliae*, the *Wor1* ortholog *VdSge1* is required for virulence on tomato [55]. Gene deletion mutants also possess developmental abnormalities associated with hyphal growth and reduced conidiation. Nine in planta highly expressed candidate effector genes [64] were examined for evidence of differential expression between the WT and *vdsge1* mutants. Six of these were reduced in expression, whereas two demonstrated increased expression in the *vdsge1* mutants [55]. This suggests that VdSge1 may function as both a positive and negative regulator of different subsets of effector genes in *V*. *dahlia* [55]. Interestingly, *VdSge1* is required for full expression of the *Ave1*. *Ave1* encode a small and Cys-rich effector that activates the host resistance response mediated by the Ve1 immune receptor [65]. It is required for full virulence on tomato plants that lacked *Ve1* [65].

*C*. *fulvum* mutants deleted in *CfWor1* demonstrated abnormal hyphal growth, abolished conidiation, and reduced virulence on susceptible tomato [57]. The expression of *Avr9*, *Avr2*, *Avr4*, *Avr4E*, and *Ecp6* were all highly reduced, particularly during the early stage of infection. Avr2 is an inhibitor of several plant Cys proteases that are required for a basal response but is recognised by the Cf-2 immune receptor [66,67]. Avr4 is a chitin binding protein that protects the fungus from plant chitinases but is recognised by the Hcr9-4D R gene located at the *Cf-4* locus [68,69]. Avr4E is a Cys-rich Avr recognised by the *Hcr9-4E* R gene also located at the *Cf-4* locus [70]. Ecp6 also binds to chitin oligomers released by the action of host chitinases, which in turn minimises recognition by the host [71]. Taken collectively, reduced effector expression could contribute to the reduced virulence of *cfwor1* mutants. In addition to effector gene regulation, transcriptome analysis identified down-regulated genes that are associated with transcription, translation, cell division, replication, and nucleic acid metabolism [57]. This indicates that CfWor1 functions as a global transcriptional regulator.

Inactivation of *ZtWor1* in the wheat septoria tritici blotch (STB) pathogen *Zymoseptoria tritici* resulted in mutants that were nonpathogenic [56]. Several genes that encode putative small secreted proteins (SSPs) were significantly down-regulated. It is yet to be fully determined if these SSPs function as effectors in the establishment of STB.

*Ustilago maydis* is a smut fungus that causes gall formation on maize. During this biotrophic interaction, the fungus secretes a suite of effectors to suppress host defence and allow infection [72]. Analysis of the *U*. *maydis* genome identified two predicted genes (*Pac2* and *Ros1*) that encode WOPR-like proteins. Deletion of *Pac2* had no effect virulence, but mutants that lacked *Ros1* did not form teliospores and demonstrated reduced virulence on maize [58]. Transcriptome analysis revealed that Ros1 negatively regulates the expression of 128 effector genes that are associated with biotrophic development, whereas 70 effector genes linked with late infection were up-regulated. Many of these genes that code for effector-like molecules have not been fully characterised with the exception of *See1*, *Cmu1*, and *Pit2* [58,73]. *Cmu1* encodes a chorismate mutase that interferes with the plant salicylic acid level [74]. Pit2 is an inhibitor of apoplastic maize Cys proteases CP1and CP2 [75]. Surprisingly, the expression *Cmu1* and *Pit2* is negatively regulated by Ros1 considering that both effectors suppress the host defence response. On the other hand, See1 is an effector that facilitates tumour expansion [76] and was shown to be positively regulated by Ros1.

### Fork head

The fork head domain is a conserved helix-turn-helix DNA binding domain with a “winged” helix structure [77]. Zahiri et al. [78] examined the role of a gene encoding a fork head TF named *Fox1* in *U*. *maydis*. Deletion of *Fox1* resulted in reduced virulence and impaired tumour formation on maize. Furthermore, Fox1 functions as a positive regulator of six candidate effector genes. It is not known whether these effector genes play a role in virulence individually. Interestingly, Ros1 negatively regulates *Fox1* expression [58].

## Regulation by chromatin modification

*L*. *maculans* possesses a highly compartmentalised genome comprising of guanine-cytosine (GC)–and adenine-thymine (AT)–rich blocks. The latter are enriched in transposable elements and genes that code for SSPs such as effectors [79]. It is hypothesised that *L*. *maculans* possesses a “two-speed” genome in which AT-isochores drive effector gene diversification for adaptation through transposon-assisted mobilisation followed by repeat-induced point mutations [80,81]. Genes that code for known Avr effectors are poorly expressed under in vitro conditions, but expression dramatically increases during host infection [79]. It was hypothesised that these AT-isochores are associated with heterochromatin regions and subjected to epigenetic modification [82]. Silencing of two genes, *LmHP1* and *LmDIM5*, that code for chromatin-modifier proteins resulted in the derepression of *AvrLm1* and *AvrLm4-7* expression during axenic growth [82]. Expression of *AvrLm1* and *AvrLm4-7* in the mutants remained similar to the WT during primary infection. Chromatin immunoprecipitation assays demonstrated that LmHP1 and LmDIM5 are involved in the formation of heterochromatin at the *AvrLm1* and *AvrLm4-7* loci in *L*. *maculans* [82]. This suggests that effector genes undergo chromatin-mediated repression that is lifted during infection.

## Regulation by effector epistasis

Epistasis can be broadly defined as interactions between genes. This can result in masking the effect of genetic variation in one gene by variation in a different gene [83,84,85]. The *P*. *nodorum*—wheat pathosystem is unique in that effector—effector epistatic interaction has been described in several studies [39,86,87,88]. However, the mechanisms accounting for epistasis between effector—host dominant susceptibility gene interactions are largely unknown. The suggested mechanisms of epistasis include differences in effector expression level, host gene action, and/or cross-talk among pathways that are associated with effector recognition [86]. In interactions with both SnTox1-*Snn1* and SnTox3–*Snn3* present, the SnTox3-*Snn3* interaction is suppressed [88]. However, when *SnTox1* was deleted, the SnTox3–*Snn3* interaction was detected during infection of a wheat mapping population using quantitative trait locus mapping. *SnTox1* deletion resulted in the up-regulation of *SnTox3* expression. The increased expression of *SnTox3* upon removal of *SnTox1* explains, in part, the mechanism of the epistatic interaction [88]. A further complication is that PnPf2 is dominant over the SnTox1 negative regulation of *SnTox3* expression. Deletion of *SnTox1* in the *pnpf2* background did not induce *SnTox3* expression [32].

Evidence of NE epistasis involving the ToxA–*Tsn1* interaction was recently observed in *P*. *tritici-repentis* [89]. When *ToxA* was deleted in *P*. *tritici-repentis*, an increase in virulence was observed on some *ToxA*-sensitive wheat cultivars as measured by an increase in tissue chlorosis. Furthermore, *ToxA* deletion has unmasked evidence of a novel chlorosis-inducing factor that was previously suppressed in the presence of *ToxA* [89,90]. This indicates that *ToxA* possess an epistatic role in effector regulation by masking the effect of other effectors.

A similar phenomenon was also observed in *L*. *maculans* where *Rlm3*-mediated resistance through recognition of the recently identified Avr protein AvrLm3 was suppressed by AvrLm4-7 [44]. *AvrLm4-7* does not influence the expression of *AvrLm3* nor do their proteins physically interact in a yeast 2-hybrid assay. Hence, the exact mechanism of suppression is unknown at this stage.

## Regulation by cellular trafficking

Recently, a cytological approach was taken to examine organelle trafficking in *U*. *maydis* during early infection [91]. It was observed that early endosomes (EEs) transmit signals from the hyphal tip to the fungal nucleus. EE motility triggered the expression of *Cmu1*, *Pit2*, and *Pep1*. The latter encodes a secreted effector that suppresses plant immunity by inhibiting host peroxidase activity [92]. These effectors are then delivered to the hyphal tip to supress host defence. Interestingly, the Crk1 mitogen-activated protein kinase is localised in the EEs during retrograde signalling to the nucleus. Crk1 is essential for mating and virulence of *U*. *maydis* on maize [93]. Surprisingly, deletion of *Crk1* resulted in the up-regulation of *Cmu1*. It was hypothesised that Crk1 may interact with an unknown phosphatase bound to the EE to coordinate effector expression and secretion [91].

## Conclusions and future prospects

Effectors function as key determinants in fungal pathogenicity. This is also dictated by the presence or absence of resistance or susceptibility genes in the host. As a result, effectors have been exploited as tools for plant breeding to improve disease resistance [7]. However, identification of signals or signalling pathways that regulate effector gene expression offers another dimension to formulate novel crop protection strategies. There is great promise in the use of small interfering RNA to inhibit fungal gene expression through superficial application or host-induced gene silencing [94,95,96]. In addition to potential crop protection benefits, efforts to understand effector regulation in fungal pathogens have already uncovered novel aspects of effector biology. We anticipate that these recent advances will stimulate further studies in the regulatory aspect of effector biology.
